# Benefits and Risks of Antiviral Treatment during Pregnancy in Patients with Chronic Hepatitis B

**DOI:** 10.3390/jcm10112320

**Published:** 2021-05-26

**Authors:** Yoon Seok Lee, Soo Min Bang, Young-Sun Lee

**Affiliations:** Division of Gastroenterology and Hepatology, Department of Internal Medicine, Korea University College of Medicine, Seoul 08308, Korea; seok7288@korea.ac.kr (Y.S.L.); soomin1987@naver.com (S.M.B.)

**Keywords:** antivirals, hepatitis B virus, mother-to-child transmission, pregnancy, tenofovir, safety

## Abstract

Hepatitis B virus (HBV) is a main cause of chronic liver disease worldwide and can lead to severe liver diseases. The World Health Organization has planned to eliminate viral hepatitis, including hepatitis caused by HBV and hepatitis C virus, by 2030. As mother-to-child transmission (MTCT) of HBV is a main cause of chronic HBV infection, MTCT prevention is the main target to reduce the risk of chronic HBV infection and eliminate the disease. Recent clinical trials and meta-analyses found that antiviral therapy could prevent MTCT effectively in mothers with ≥200,000 IU/mL of HBV DNA, in combination with serial vaccination and hepatitis B immune globulin administration in infants. Despite the preventive role of antivirals for MTCT of HBV, there are several concerns regarding antiviral therapy with respect to the safety of the mother and fetus during pregnancy. This review summarizes the benefits and risks of antiviral treatment during pregnancy in women with chronic HBV infection.

## 1. Introduction

The global prevalence of hepatitis B virus (HBV) was 3.5% in 2015 [1]. Approximately 257 million people, including 65 million women of childbearing age, were infected with HBV, and 884,000 patients died because of HBV-related diseases [1,2,3]. Therefore, the World Health Organization declared the elimination of viral hepatitis by 2030, including hepatitis caused by HBV and hepatitis C virus, as a public health goal. Owing to the hepatitis B vaccine, the number of newly HBV infected patients has decreased. However, there were still approximately 4.7 million new patients with HBV infection in 2015 [4]. The main cause of HBV transmission is mother-to-child transmission (MTCT) during pregnancy and the peripartum period [5]. This is because 90% of the infections in infants become chronic; otherwise only 6% of infections in people over the age of 5 years become chronic [1]. Therefore, preventing MTCT of HBV is the main target to reduce the risk of chronic hepatitis B (CHB) and eliminate the disease. Using an immunoprophylactic strategy of serial vaccination and hepatitis B immune globulin (HBIG) administration to children within 12 h of delivery, the rate of MTCT of HBV has decreased by approximately 90% in infants born to mothers with a high viral load [6,7]. Nevertheless, infants from hepatitis B e antigen (HBeAg)-positive mothers or mothers with a high viral load still have a risk of acquiring the infection by MTCT [8,9]. Therefore, attempts have been made to prevent the MTCT of HBV by administering antiviral drugs to pregnant CHB patients HBeAg-positive and with a high viral load. However, there are several concerns about antiviral therapy in pregnancy. In this review, we discuss the benefits and risks of antiviral treatment during pregnancy to prevent MTCT of HBV.

## 2. Mechanism of MTCT of HBV

MTCT of HBV can be classified into two categories: intrauterine transmission (IUT) and perinatal transmission. Although the placenta protects against the transmission of the hepatitis B surface antigen (HBsAg) and viral particles, several mechanisms allow HBV transmission from the mother to the fetus before labor. HBV-infected oocytes, transmission through peripheral blood mononuclear cells, and placental infection could be rare causes of IUT [10,11,12]. IUT is mainly caused by placental injury, allowing HBV to be transmitted through the placental barrier to infants [13,14]. Thus, prenatal invasive tests, such as amniocentesis, can result in IUT. Amniocentesis performed in CHB mothers with high viral load (HBV DNA ≥ 7.0 log_10_ IU/mL) increased the rate of MTCT significantly in a case-control study [15]. Therefore, the risk of MTCT of HBV with amniocentesis should be considered with a risk–benefit assessment in mothers with high HBV DNA [16].

The exact proportions of intrauterine and perinatal transmission in MTCT of HBV are unknown, but perinatal transmission is known to be the key form of MTCT of HBV [8]. Fetal trauma during labor, micro-circulation between maternal and fetal blood, and contact with HBV-containing vaginal fluid/epithelium could be possible mechanisms of HBV transmission during the perinatal period [17]. Maternal HBV DNA level is the most significant single risk factor for MTCT of HBV, and the transmission risk increases in mothers with a higher viral load [8,9]. The risk of MTCT with a maternal HBV DNA level of ≥200,000 IU/mL is higher than that with a maternal HBV DNA level of <200,000 IU/mL [18,19,20]. HBeAg-positive status and high serum HBsAg level are also related to an elevated risk of MTCT of HBV, and they can be substituted for HBV DNA to estimate MTCT risk in areas where HBV DNA testing is not available [21,22,23,24]. In general, HBeAg-positive mothers have a higher HBV DNA level than HBeAg-negative mothers. Children from HBeAg-positive mothers have a higher risk of infection compared with those from HBeAg-negative mothers [22]. In a meta-analysis of 66 studies, the pooled analysis showed that the sensitivity of HBeAg status for prediction of HBV DNA levels of ≥200,000 IU/mL was 88.2% (83.9–91.5%), and the specificity was 92.6% (90.0–94.5%) [23]. HBsAg quantification was reported to predict infection in infants in a study on 568 HBsAg-positive mothers in Taiwan [21]. HBeAg can pass the placenta and is transmitted to the fetus during the intrauterine period. In HBeAg-positive mothers, HBeAg that crosses the placenta can weaken the immune response against HBV in children [25]. Likewise, co-inhibitory signaling by macrophages may reduce antiviral effects in children exposed to HBV in the uterus [26].

Cesarean section was shown to reduce the risk of HBV MTCT compared to normal vaginal delivery in a few studies [27,28]. However, no randomized controlled trials (RCTs) have proven that cesarean section reduces the risk of MTCT. Consequently, cesarean section is not indicated to prevent MTCT of HBV.

## 3. Immunization Strategy to Prevent MTCT of HBV

In addition to serial vaccination of HBV, administration of HBIG is critical for preventing MTCT. The immune barrier formed by HBIG provides protection against MTCT of HBV with safety and efficacy [29]. In a RCT of HBeAg-positive mothers in Hong Kong, the rates of MTCT of HBV without vaccination, with only vaccination, and with vaccination + HBIG administration were 73.2%, 21.0%, and 6.8%, respectively [30]. Furthermore, immunization of infants against HBV prevents the development of HCC in adults [31]. The HBV vaccine and HBIG should be injected within 12–24 h of delivery [1,16]. Delayed administration is associated with immunoprophylaxis failure [32]. However, global coverage of hepatitis B vaccine was 39% in 2015 [1]. Easy access to the hepatitis B vaccine is required to reduce risk of MTCT of HBV and to accomplish the goal of HBV elimination.

## 4. Antiviral Treatment during Pregnancy for the Prevention of MTCT of HBV

Despite adequate immunoprophylaxis with HBIG administration and HBV vaccination, MTCT can still occur in some cases, especially in infants born to mothers with a high HBV viral load (>200,000 IU/mL) or with HBeAg-positive status [9,22,33]. Most CHB patients of childbearing age are in the immune tolerant phase [34]. Pregnant women in the immune tolerant phase have a high HBV DNA titer in the plasma, resulting in an elevated risk of viral transmission by the exposure of infants to HBV during delivery [35]. Therefore, additional intervention with antiviral treatment during pregnancy has been investigated in women with a high risk of MTCT [36].

In terms of efficacy, perinatal antiviral therapy in pregnant CHB patients with high viral load was related with a significant reduction in the rate of HBsAg or HBV DNA positivity in the infant at 6–12 months [37]. According to a cost-effectiveness analysis, an antiviral prophylaxis strategy proved cost-effective compared with the strategy used in the United States (universal HBsAg screening in pregnant women; infants born to CHB patients receive the HBV vaccine and HBIG within 12 h of birth, while all other infants receive the HBV vaccine during hospitalization) [38]. The history of antiviral treatment during pregnancy in women with CHB has been similar to the history of antiviral treatment in patients with CHB. Lamivudine, telbivudine, and tenofovir disoproxil fumarate (TDF) were studied in terms of preventing MTCT of HBV during pregnancy. Lamivudine, the first oral nucleoside analog, was initially investigated for the prevention of MTCT of HBV [39]. Lamivudine reduced the risk of MTCT of HBV as reported by five RCTs (relative risk (RR) = 0.29, 95% confidence interval (CI) 0.15–0.56) [37]. The administration of telbivudine, an oral nucleoside analog, was found to be safe during pregnancy [40]. Telbivudine also effectively reduced the risk of MTCT of HBV as per four RCTs (RR = 0.23, 95% CI 0.10–0.52) [37]. Although both lamivudine and telbivudine showed effectiveness in preventing MTCT of HBV, they are not currently used as first-line therapies in CHB patients because of their low potency and low barrier of resistance. TDF is the preferred first-line antiviral agent, as its potency and resistance barrier are higher than those of lamivudine and telbivudine [1,16,41]. In the first open-label RCT on this topic conducted by Pan et al. in 2016, TDF significantly reduced the MTCT of HBV in both an intention-to-treat analysis (5% in the TDF group vs. 18% in the control group) and per-protocol analysis (0% in the TDF group vs. 7% in the control group, P = 0.01) [42]. This study included 200 mothers HBeAg-positive and with HBV DNA >200,000 IU/mL. However, a double-blind RCT from Thailand did not show any significant statistical difference in the MTCT of HBV between the placebo control group and TDF group [43]. The insufficient efficacy of TDF in this study might have been due to the inclusion of mothers with a low viral load (11% of participants, HBV DNA <200,000 IU/mL) and the administration of five doses of HBIG. Nevertheless, several meta-analyses have shown significant efficacy of TDF in the prevention of MTCT of HBV [44,45,46]. In the meta-analysis of five RCTs, the pooled odds ratio of MTCT of HBV based on HBsAg positivity in infants was 0.10 (95% CI 0.03–0.35) [46]. Using two studies with a low risk of bias, the odds ratio in the intention-to-treat analysis was 0.53 (95% CI 0.13–2.17), whereas the odds ratio in the per-protocol analysis was 0.10 (95% CI 0.01–0.77) [45]. Since lamivudine and telbivudine have a lower genetic barrier than TDF, using lamivudine or telbivudine during pregnancy may increase the risk of drug resistance, and thus, TDF is the preferred antiviral agent during pregnancy [47,48]. Although there is no RCT for TAF during pregnancy for preventing MTCT of HBV, one multicenter prospective observational study reported that TAF is effective for preventing MTCT of HBV (0% of MTCT rate). [49] The efficacy of antivirals for preventing MTCT of HBV is summarized in Table 1.

The appropriate timing for starting antiviral treatment remains unknown. Antiviral treatment was initiated at 24–32 weeks of gestation in most clinical trials. For pregnant CHB patients with serum HBV DNA levels >200,000 IU/mL, the American Association for the Study of Liver Disease (AASLD) recommends starting TDF administration in the second trimester, and the European Association for the Study of the Liver (EASL) recommends starting at 24–28 weeks of gestation [16,50]. A more recent post hoc meta-analysis of 14 studies (n = 2, TDF; n = 10, telbivudine; and n = 2, lamivudine) found that initiation of antiviral treatment in the second trimester showed a higher efficacy in preventing the MTCT than starting treatment in the third trimester (OR 0.23; 95% CI 0.09–0.59) [46]. Therefore, the timing of antiviral treatment initiation should not go beyond the third trimester, and it is preferable to start antiviral treatment at 24-28 weeks of gestation. In particular, beginning antiviral therapy early in the second trimester may be considered for pregnant women with CHB with a very high viral load (>8 log_10_ IU/mL) to secure time for the HBV load to fall below 200,000 IU/mL at the time of birth [65]. Pregnant women who are in the immune-active phase of CHB should be treated with antiviral agent if they meet standard indication [66]. If treatment is performed with another antiviral drug, changing to TDF should be considered. CHB patients with cirrhosis and their infants have an increased risk of obstetric complications [67]. In addition, cirrhotic CHB mothers can develop decompensation during pregnancy. Therefore, clinicians should manage these patients more carefully, including antiviral therapy in case of indication. HIV co-infection in pregnant women does not increase the risk of MTCT in the setting of an antiretroviral therapy that contains tenofovir and/or lamivudine [68].

**Figure 1:**
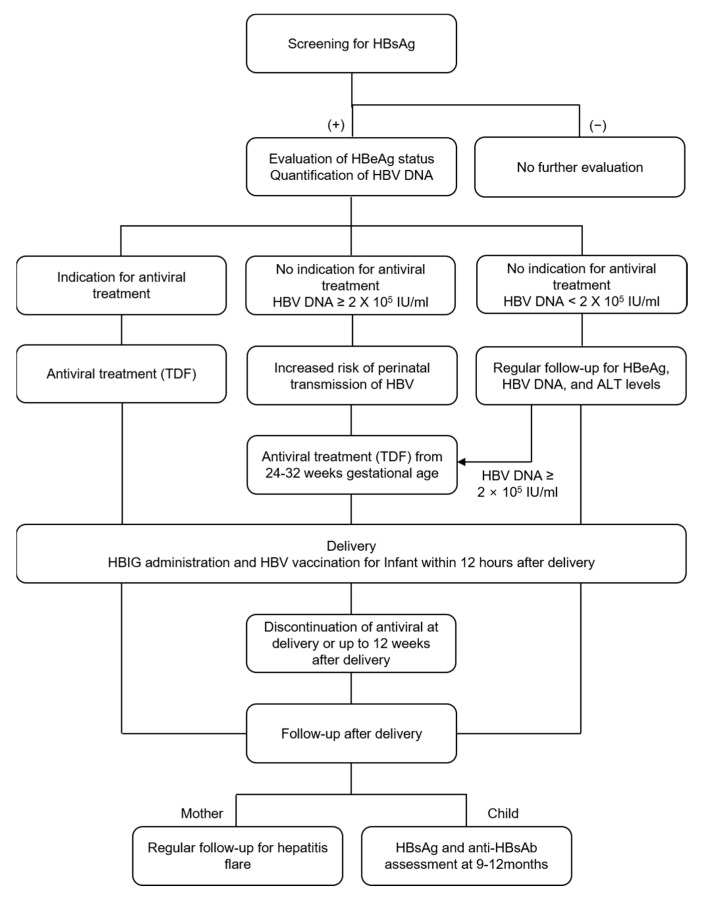
HBV screening and prevention of HBV MTCT in pregnant women. MTCT, mother-to-child transmission; HBsAg, hepatitis B surface antigen; HBeAg, hepatitis B envelope antigen; HBV DNA, hepatitis B virus DNA; IU, international units; TDF, tenofovir disoproxil fumarate; ALT, alanine aminotransferase; HBIG, hepatitis B immune globulin.

The timing of discontinuation is also not well defined in cases in which antiviral treatment is initiated only for the prevention of MTCT. The AASLD stated that in women who receive antiviral treatment for preventing MTCT of HBV, antiviral treatment could be stopped immediately after delivery or up to 4 weeks postpartum. The EASL suggested that TDF should be continued for up to 12 weeks after procreation [16]. The treatment efficacy was not much different according to the timing of antiviral discontinuation (at delivery or at 4–8, 12, or 24 weeks postpartum) [46]. Clinicians should carefully check whether hepatitis flares occur in mothers after the discontinuation of antiviral agents up to 12 weeks after delivery. The suggested management algorithm for pregnant CHB patients is summarized in Figure 1.

## 5. Maternal Risks Associated with Antiviral Treatment for Preventing MTCT

Higher C-section rates, preterm labor, postpartum hemorrhage, and postpartum hepatitis B flares are predictable maternal complications of antiviral treatment. However, the safety of telbivudine, lamivudine, and TDF has been identified in large-scale systematic reviews, with no increase in the occurrence of adverse maternal outcomes [37,46]. Mothers with CHB are usually in the immune tolerant phase during pregnancy, showing an increase in HBV DNA levels and a decrease in alanine aminotransferase (ALT) levels due to weakening of the immune response during the early stage of pregnancy. In the late period of pregnancy, HBV DNA levels decrease and ALT levels increase secondarily to the restoration of immune response. In the case of antiviral cessation, the risk of hepatitis flares is high, and close monitoring is required. The definition of a hepatitis flare varies across the literature, but a significant increase in serum ALT levels from the baseline level or upper normal limit is generally defined as a flare. Postpartum hepatitis B flares are associated with immunological changes in pregnant women. The balance between the Th1 response and Th2 response is biased toward the Th2 response during pregnancy [69]. Consequently, the maternal immune system is suppressed during pregnancy and is reactivated after delivery. This alteration allows the fetus to escape maternal immune invasion [69,70]. However, the reconstitution of maternal immunity after delivery causes hepatitis flares. There have been concerns that the discontinuation of antiviral treatment may exacerbate hepatitis flares. Although there was an increased tendency toward the development of postpartum hepatitis flares after the discontinuation of antiviral agents in the antiviral-treated group compared to the non-antiviral-treated group, the severity of hepatitis was not different, and most patients showed resolution with or without antiviral treatment [71]. The most recent meta-analysis on this topic showed that lamivudine, telbivudine, and TDF can be used safely with no increase in the risk of postpartum hepatitis flares after treatment discontinuation [46]. However, the risks of complications, hepatic decompensation, and mortality are increased in mothers with CHB with advanced fibrosis or liver cirrhosis. Therefore, antiviral treatment should be continued in such patients [67].

Pregnancy is a contraindication to pegylated interferon (Peg-IFN) administration [72]. If women treated with Peg-IFN have an unexpected pregnancy, treatment discontinuation should be considered. If the treatment needs to be continued, then TDF is the preferred alternative [50].

## 6. Fetal Risks Associated with Antiviral Exposure during Pregnancy

Antiviral medication should be prescribed during pregnancy with caution because such medications affect the mother and may cause fetal deformities or abortion. The currently approved oral antiviral agents are nucleos(t)ide analogs, and these drugs have the potential for inducing mitochondrial toxicity that can lead to adverse fetal outcomes [73]. As per the previous Food and Drug Administration (FDA) pregnancy class definitions, lamivudine and entecavir were classified as class C medications, indicating that these are potentially harmful drugs, and well-controlled human data regarding these drugs are lacking; in contrast, telbivudine and TDF were classified as class B medications, indicating no risk as per animal studies but a lack of data from well-controlled human studies. The safety of telbivudine, lamivudine, and TDF has been identified in large-scale meta-analyses without increasing in the occurrence of adverse maternal or fetal outcomes [37,46]. Since the Pregnancy and Lactation Labeling Rule replaced the FDA pregnancy categories, the risks associated with medication use during pregnancy are assessed individually [74]. According to the Antiretroviral Pregnancy Registry (APR), which collects and evaluates data on the adverse outcomes of pregnancy after exposure to antiretroviral drugs, there was no significant difference in birth defect rates between the lamivudine-treated (3.12%), telbivudine-treated (1.18%), and TDF-treated (2.37%) groups and their respective control cohorts [59]. No major congenital anomalies have been reported with respect to treatment with lamivudine, telbivudine, or TDF.

However, there are several safety issues associated with fetal exposure to antiviral agents during pregnancy. Exposure to both lamivudine and zidovudine caused prematurity, anemia, and neurological symptoms due to mitochondrial dysfunction [60,61]. There are concerns regarding renal and bone toxicity due to TDF [62]. As TDF is present at concentrations of >100 ng/mL in fetal blood [75], TDF exposure during the fetal period might affect renal function and growth in children. One study reported that TDF-exposed infants showed lower bone mineral content and growth retardation in mothers who received TDF treatment for human immunodeficiency virus (HIV) infection [63,64]. However, another meta-analysis showed no difference in growth or bone abnormalities between TDF-exposed and non-TDF-exposed infants of mothers with HIV infection [76]. According to a recent prospective study that followed-up infants exposed to TDF from gestational age of 30–32 weeks and non-TDF-exposed infants, clinical parameters including body weight, height, bone development, and renal function were not significantly different between the two groups for up to 6–7 years [77]. Although tenofovir alafenamide (TAF) is the preferred agent for CHB due to the low risk of renal and bone-related adverse effects, safety studies on TAF in the context of pregnancy are limited.

Other factors related to fetal safety are also of concern for antiviral treatment during pregnancy. However, several meta-analyses have reported that there were no significant increases in the occurrence of other outcomes related to fetal safety, such as fetal death, preterm birth, low birth weight, fetal infection, and fetal jaundice [44,45,46]. Fetal safety concerns due to exposure to antiviral agents during pregnancy are summarized in Table 1.

## 7. Postpartum Follow-Up of the Mother and Child

As flare occurrence was not influenced by the discontinuation of antiviral agents (at delivery or at 4–8, 12, or 24 weeks postpartum) [46], antivirals may be discontinued after delivery in mothers who were treated with antivirals to prevent MTCT. Although the timing of discontinuation is not well defined for patients in whom antiviral treatment is initiated only for the prevention of MTCT, most international guidelines recommend that antiviral treatment could be discontinued up to 12 weeks after delivery, as described above [78]. After the discontinuation of antivirals, clinicians should carefully monitor mothers for the occurrence of hepatitis flare.

Children born to mothers with CHB are recommended to be tested for HBsAg and hepatitis B surface antibody (anti-HBsAg) at 9–12 months after birth to identify successful prevention of MTCT of HBV and the production of anti-HBsAg antibody [79]. As HBsAg may appear due to HBV vaccination and anti-HBsAg antibodies can be detected as a result of HBIG administration at birth, testing in children before 9 months of age is not recommended [65]. If children have not acquired immunity against HBV infection and show no HBV infection, they should receive the HBV vaccine again. Children with HBV infection transmitted from the mother are recommended to undergo regular monitoring of HBeAg, ALT, and HBV DNA levels, despite the low risk of developing liver cirrhosis or HCC at this early age [80]. Although data are limited, children also require HCC surveillance, with regular alpha-fetoprotein measurement and sonography, especially children showing HBeAg seroconversion and cirrhosis [81].

## 8. Breastfeeding in Women Who Received Antiviral Treatment

As HBV transmission through breast milk is extremely rare, breastfeeding is not restricted in mothers with CHB [82]. However, drugs may be transferred from the mother to the infant during breastfeeding, which may be harmful to the infant. In addition, some maternal medications could be hazardous for breastfeeding infants, even though these agents are safe during pregnancy [83]. Therefore, the safety of antiviral treatment during breastfeeding is an important issue. After absorption in the gastrointestinal tract, TDF exists in the blood in the form of tenofovir, and a small amount of tenofovir is excreted through breast milk [84,85]. Therefore, previous AASLD guidelines addressed the unknown risk of low-level exposure of antivirals in the child via breastfeeding by mothers treated with TDF [66]. However, tenofovir, excreted through breast milk, is rarely absorbed by infants [75]. Thus, the tenofovir concentration in infant plasma is extremely low [86]. Breastfeeding after adequate immunoprophylaxis is not a cause of MTCT of HBV, and furthermore, antiviral treatment during breastfeeding is safe [75,87]. The latest international guidelines consistently allow breastfeeding of newborns regardless of maternal HBV status and treatment with TDF [16,50].

## 9. Future Perspectives

Although TDF is safe in terms of renal function and growth in infants, there is a limitation in that these results did not originate from a randomized controlled study [77]. In addition, while safety has been reported in infants, the long-term safety of TDF exposure during the fetal period should be evaluated in studies with a long-term follow-up period. The role of TAF in preventing MTCT of HBV and the safety of TAF during pregnancy are important issues that need to be studied. Although TAF showed meaningful antiviral effects against HIV and showed safety in both the mother and child [88], the APR reported that TAF use was associated with a higher rate of birth defects due to TAF exposure in the first trimester [59]. Therefore, further studies are required to assess the suitability of the clinical application of TAF for preventing MTCT of HBV. One multicenter prospective observational study reported that TAF is effective for preventing MTCT of HBV (0% of MTCT rate) and safe during pregnancy (no birth defect) [49]. Recently, a prospective study has been conducted in China (NCT04237376) for evaluation of the efficacy and safety of TAF for preventing MTCT of HBV. If the study is completed successfully, the efficacy and safety of TAF in preventing MTCT of HBV can be identified.

## 10. Conclusions

In addition to sequential vaccination and administration of HBIG, antiviral treatment in pregnant CHB patients with high HBV DNA titers (≥200,000 IU/mL) can effectively prevent MTCT of HBV. Among the currently available antiviral agents, TDF is safe and effective for prevention of MTCT of HBV. If the mother’s serum HBV DNA level is >200,000 IU/mL, clinicians should consider administering TDF to prevent MTCT of HBV from 24–28 weeks of pregnancy to 2–12 weeks after delivery. This strategy is expected to contribute greatly to the elimination of HBV infection as a public health issue.

## Figures and Tables

**Table 1:** Summary of antiviral agents used for HBV infection during pregnancy.

	Lamivudine	Telbivudine	TDF	TAF
Preferred initial therapy for CHB patients [16,50]	No	No	Yes	Yes
Resistance rate [51,52]	70% after 6 years	17% after 2 years	0% up to 6 years	0% after 2 years
Effectiveness in terms of the prevention of MTCT of HBV	67–78% [37,53,54,55]	77–94% [37,54,56,57,58]	77–90% [23,44]	100% [49]
Rate of postpartum flares after treatment cessation [46]	13.2%	6.3%	7.9%	NA
Previous FDA category	C	B	B	NA
Birth defect rates after exposure during the first trimester [59]	3.11% (168/5398)	1.18% (3/254)	2.39% (105/4388)	4.38% (19/434)
Other fetal safety concerns	Prematurity, anemia, and neurological symptoms due to mitochondrial dysfunction (combination with zidovudine) [60,61]	Not reported	Renal toxicity, growth retardation, and bone-related toxicity [62,63,64]	Insufficient data are available.

TDF, tenofovir disoproxil fumarate; TAF, Tenofovir alafenamide; CHB, chronic hepatitis B; MTCT, mother-to-child transmission; FDA, Food and Drug Administration.

## Data Availability

Not applicable.
